# A comparison of the chromosome G-banding pattern in two *Sorex* species, *S. satunini* and *S. araneus* (Mammalia, Insectivora)

**DOI:** 10.3897/CompCytogen.v6i3.3019

**Published:** 2012-08-20

**Authors:** Yuriy M. Borisov, Victor N. Orlov

**Affiliations:** 1 Severtsov Institute of Ecology and Evolution, Russian Academy of Sciences, Leninskii pr., Moscow, 119071 Russia

**Keywords:** G-banding, common shrew, *Sorex araneus*, *Sorex satunini*, karyotype, phylogeny

## Abstract

The G-banded karyotype of *Sorex satunini* was compared with the karyotype of *Sorex araneus*. Extensive homology was revealed. The major chromosomal rearrangements involved in the evolutionary divergence of these species have been identified as centric fusions and centromeric shifts. From the known palaeontological age of *Sorex satunini* it is obvious that the vast chromosomal polymorphism of the *Sorex araneus* group originated during the middle Pleistocene.

## Introduction

Within the genus *Sorex* Linnaeus, 1758, the *Sorex araneus* group includes eight species characterized by the sex chromosome complex XY_1_Y_2_ (Zima et al. 1998). Some of them were raised to species status on a karyological basis. Two species from this group, the common shrew *Sorex araneus* Linnaeus, 1758 and the Caucasian shrew *Sorex satunini* Ognev, 1922 can be defined as cryptic species that are virtually impossible to distinguish by morphological (cranial) characters (Sokolov and Tembotov 1989).

The common shrew is widely distributed in Europe and Asia up to as far east as Lake Baikal, and the Caucasian shrew is known to be present in the Caucasus and in the northern parts of Asia Minor (Sokolov and Tembotov 1989, Bukhnikashvili and Kryštufek 2008). In the North Caucasian plains, this species is contiguous with the common shrew (the chromosomal race Neroosa) (Stacheev et al. 2010).

The common shrew displays phenomenal variability of the autosomal complement (Wójcik et al. 2002). The Caucasian shrew is monomorphic and can be reliably identified by means of conventionally stained karyotype (Kozlovsky 1973, Sokolov and Tembotov 1989, Macholán 1996). Macholán (1996) recognized in G-banded metaphases of the *Sorex satunini* the autosomes *af, bc* and *tu*, which are invariantly present in the common shrew karyotype. The presence of these Robertsonian fusions in the *Sorex satunini* corroborates the findings of Zagorodniuk and Khazan (1996) who described the arm combinations of autosomes *af, bc, gh, ik, jn, lo*, and *tu* in the karyotype of a single female from Kobi (Georgia).

From the plain between the Kuban and Don rivers we described a new subspecies of the Caucasian shrew *Sorex satunini tembotovi* Orlov, Balakirev, Borisov, 2010(Orlov et al. 2010) that differs from the subspecies *Sorex satunini armenica* Sokolov et Tembotov, 1989 and *Sorex satunini stavropolica* Sokolov et Tembotov, 1989.

In this study the karyotypes of *Sorex satunini tembotovi* and *Sorex araneus* (chromosome race Moscow) were examined and compared.

## Material and methods

Three females and four males of *Sorex satunini* were captured in the valley of the Beisoog River (45°40'N, 39°41'E), 90 km N of the Krasnodar city in June 2009. Two shrews of the race Moscow (male and female) were captured in Moscow vicinity.

Mitotic chromosome spreads were prepared in the field conditions from bone marrow and spleen cells using the air-drying technique after fixation with methanol and glacial acetic acid. For G-banding, the slides were treated with trypsin solution according to Seabright (1971). Chromosome nomenclature used follows Searle et al. (2010).

## Results and comments

The karyotype of *Sorex satunini* consists of 24–25 chromosomes. The sex chromosomes are a large metacentric X, a small acrocentric Y_1_, and a medium-sized Y_2_. Of 11 autosomal pairs, only a single pair of small chromosomes is acrocentric, all other autosomes are biarmed. Such a karyotype has been described for many populations from the North Caucasian and Transcaucasian regions (Kozlovsky 1973, Sokolov and Tembotov 1989, Macholán 1996).

**Figure 1.**
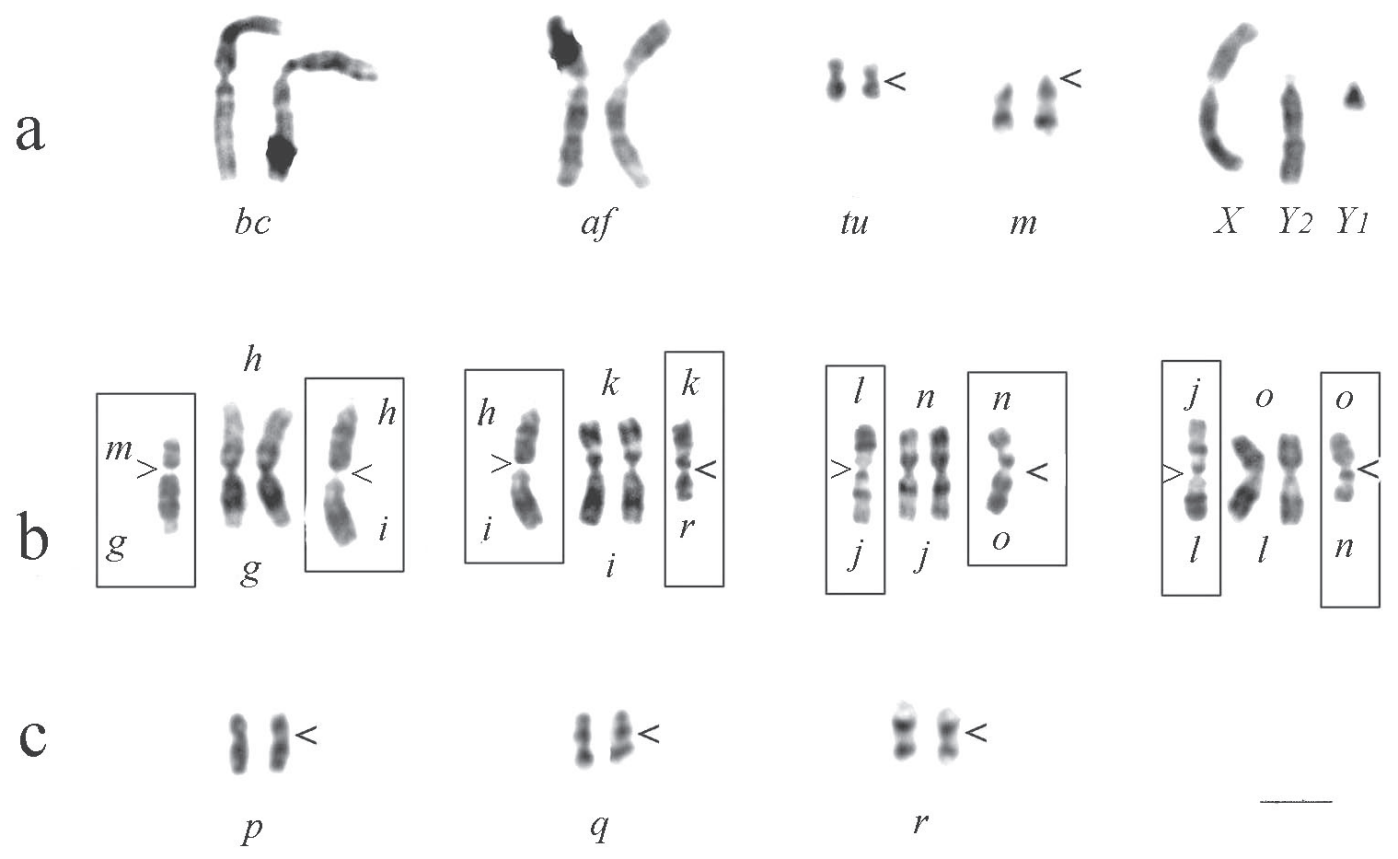
The G-banded karyotype of *Sorex satunini* (male) in comparison with the karyotype of *Sorex araneus* (the race Moscow, male). The chromosomes of the race Moscow are given in the frameworks. Some chromosomes are identical (**a**), the others are different because of the arms involved in different fusions (**b**) or because of the centromeric shift (**c**). Centromere position is indicated by “<”. Bar = 3 µm.

The Caucasian shrew has the following chromosome formula: XX / XY_1_Y_2_, *af, bc, gh, ik, jn, lo, tu, m, p, q, r, tu*. The comparison of the G-banded metaphase chromosomes of *Sorex satunini* and of *Sorex araneus* (the race Moscow) is presented in Fig. 1. This comparison revealed a considerable homology between individual chromosomal arms. Identical banding patterns and centromeric positions were found in two large biarmed autosomes *af* and *bc*, in small metacentric *tu*, in acrocentric pare *m* and in the sex chromosomes (Fig. 1a). The acrocentric *m* is found in the karyotypes of the chromosomal races of *Sorex araneus* either as an individual acrocentric, or in combination with other acrocentrics.

Seven arms of *Sorex araneus*, namely *g, i, k, j, n, l*, and *o*, were also identified in the complement of *Sorex satunini*. The difference in G-banding of the arm *h* between *Sorex satunini* and other species of the *Sorex araneus* group was observed (Fig. 1b). An identical banding pattern and a different centromeric position were found in three autosomal pairs: *p, q*, and *r*, suggesting occurrence of centromeric shift. The chromosomes *p, q*, and *r* were found to be metacentric in the complement of *Sorex satunini* (Fig. 1c) and acrocentric in *Sorex araneus*.

The large biarmed chromosome *bc* was also identified in the complement of *Sorex antinorii* Bonaparte, 1840, and only in *Sorex coronatus* Millet, 1828it was substituted by *ci* (Hausser and Jammot 1974). The biarmed chromosome *lo* is found in the karyotypes of *Sorex coronatus* and *Sorex antinorii*, the biarmed chromosome *jn -* in the karyotype of *Sorex coronatus* (Brünner et al. 2002). The metacentric *ik* is known in five chromosomal races of *Sorex araneus* (Wójcik et al. 2003). The metacentric *gh* was identified only in the complement of *Sorex satunini* (Fig. 1b).

In karyotype of *Sorex coronatus* there are only two species-specific chromosome rearrangements (Rb fusions *ci* and *mp*). In karyotype of *Sorex antinorii* there are only two species-specific chromosome rearrangements, too (*hj* and *kn*). In karyotype of *Sorex satunini* there are five species-specific chromosome rearrangements (Rb fusion *gh*, centromeric shifts in the chromosomes *p, q, r*, and, probably, a paracentric inversionin the chromosomal arm *h*), i.e. *Sorex satunini* has more rearranged karyotype than the species of *Sorex araneus* group in Western Europe.

A number of chromosome rearrangements shared by *Sorex araneus*, *Sorex satunini* and *Sorex antinorii* (centric fusions *bc*), by *Sorex coronatus*, *Sorex satunini* and *Sorex antinorii* (*lo*), by *Sorex coronatus* and *Sorex satunini* (*jn*) suggest the existence of a common ancestral species in the Pleistocene of Europe analogous to the modern *Sorex araneus*.

The known paleontological age points to an early origin of *Sorex satunini*. At present, the dating of fossils confirmed by the radiocarbohydrate analysis is known only for *Sorex satunini*. These fossils, morphologically very similar to the recent *Sorex satunini* were found in the Transcaucasian region (Kudaro caves) in all layers of the middle and late Pleistocene, beginning since 0.36 Myr BP (Osipova 2006).
